# Flame Retardancy of Wood Fiber Materials Using Phosphorus-Modified Wheat Starch

**DOI:** 10.3390/molecules25020335

**Published:** 2020-01-14

**Authors:** Stefan Gebke, Katrin Thümmler, Rodolphe Sonnier, Sören Tech, André Wagenführ, Steffen Fischer

**Affiliations:** 1 Institute of Plant and Wood Chemistry, Technische Universität Dresden, 01062 Dresden, Germany; stefan.gebke@forst.tu-dresden.de (S.G.); sfischer@forst.tu-dresden.de (S.F.); 2 C2MA, IMT—Mines Alès, 6, avenue de Clavières, 30100 Alès, France; rodolphe.sonnier@mines-ales.fr; 3 Wood and Fibre Material Technology, Technische Universität Dresden, 01062 Dresden, Germany; soeren.tech@tu-dresden.de (S.T.); andre.wagenfuehr@tu-dresden.de (A.W.)

**Keywords:** wood fiber, starch derivatives, biopolymer-based flame retardants, smoldering, phosphate, carbamate, urea

## Abstract

Biopolymer-based flame retardants (FR) are a promising approach to ensure adequate protection against fire while minimizing health and environmental risks. Only a few, however, are suitable for industrial purposes because of their poor flame retardancy, complex synthesis pathway, expensive cleaning procedures, and inappropriate application properties. In the present work, wheat starch was modified using a common phosphate/urea reaction system and tested as flame retardant additive for wood fibers. The results indicate that starch derivatives from phosphate/urea systems can reach fire protection efficiencies similar to those of commercial flame retardants currently used in the wood fiber industry. The functionalization leads to the incorporation of fire protective phosphates (up to 38 wt.%) and nitrogen groups (up to 8.3 wt.%). The lowest levels of burning in fire tests were measured with soluble additives at a phosphate content of 3.5 wt.%. Smoldering effects could be significantly reduced compared to unmodified wood fibers. The industrial processing of a starch-based flame retardant on wood insulating materials exhibits the fundamental applicability of flame retardants. These results demonstrate that starch modified from phosphate/urea-systems is a serious alternative to traditional flame retardants.

## 1. Introduction

Wood fibers are one of the most common materials for heat and sound insulation based on renewable raw materials [1,2]. In contrast to most synthetic insulations, they are recyclable, compostable, non-toxic, and consume less energy in the manufacturing process [3]. But similar to petroleum-based insulation, wood fibers have a low resistance to fire [4]. Flame retardant additives are necessary for use as a material in the building sector [5]. To improve fire properties, primarily inorganic salts are used in the industry so far. These include predominantly halogen, boron, and ammonium salts. However, a lot of these currently used flame retardants have come under criticism due to ecological and toxicological concerns [5,6,7,8,9].

Various reaction systems have been examined for the synthesis of FR from biopolymers. However, many of the FR discussed can’t be obtained in large quantities, or the flame-retardant effect is usually not sufficient enough [10]. Often, complex syntheses with toxic compounds are necessary to equip the polymer with the desired properties [11,12,13]. Material incompatibilities and poor application properties limit the usability of effective systems [14,15]. In addition, extensive purification steps of the synthesis products and disposal of by-product streams make flame retardant production energy-intensive and costly. Therefore, to date there is no known commercially available flame retardant based on renewable raw materials, which can be produced and applied industrially [16].

**Figure 1:**
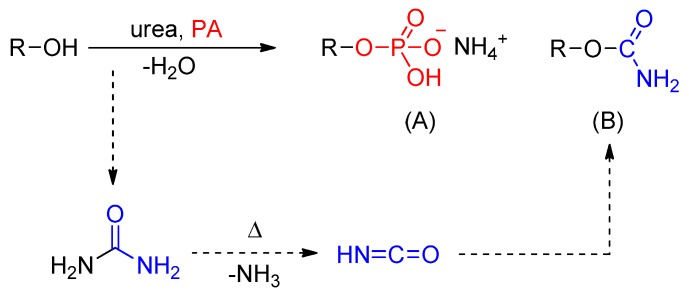
Reaction scheme for the functionalization of starch (R) with phosphate agents (PA) in urea; (**A**) esterification yielding mainly monoammonium phosphate compounds, (**B**) formation of carbamate groups by the side reaction with isocyanic acid from urea decomposition.

A promising approach is the functionalization by phosphate/urea reaction systems [17,18]. This method allows the incorporation of the fire proofing elements phosphorus and nitrogen in a one-step synthesis onto the biopolymer with available and harmless chemicals (see Figure 1). The heterogenic reaction occurs solvent-free in a urea melt with common phosphating agents (PA) like phosphoric acid (H_3_PO_4_) monoammonium phosphate (*MAP*, NH_4_H_2_PO_4_) or urea phosphate (CO(NH_2_)_2_·H_3_PO_4_). So far, mainly polysaccharides such as cellulose and starch have been functionalized [19]. The products contain primarily phosphate esters and a certain amount of nitrogen (see Figure 1).

**Figure 2:**
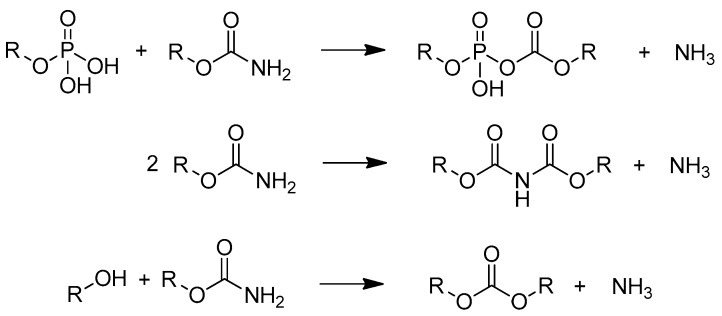
Possible condensation reactions of phosphates and carbamate groups, R = starch, based on [21,22,26].

A degree of substitution, defined as the average number of phosphate groups per anhydroglucose unit, up to 1.0 could be achieved depending on conditions like temperature, pressure, and reaction time [13,19]. This is high compared to heterogeneous reactions without urea which are in the range below 0.1 [13]. Phosphates are trifunctional groups and possess the ability to cross-link, which can lead to insoluble products with undefined structure [20]. In addition to esterification, the formation of isocyanic acid from urea decomposition during melting leads to the incorporation of carbamate groups. These are also able to crosslink by further reactions with elimination of ammonia (see Figure 2). It is known that high temperatures and long reaction times promote this kind of condensation reactions [21,22]. As side-products, unreacted urea, phosphating agents, and biuret from urea decomposition remain in the product which can be washed out by dialysis, filtration, and precipitation procedures [23,24,25].

So far, polysaccharide derivatives from phosphate/urea systems were previously discussed as water absorbing hydrogel for waste water treatment and applications in pharmacy, food industry, cosmetics, and agriculture [18,22,27]. There is little data on flame retardant additives processed and tested through this method [28]. It is largely unknown whether the additives are applicable for industrial requirements and what protective effects they have.

Thus, the current study investigates wheat starch from phosphate/urea-systems as flame retardant additive for wood fiber materials. The fire behavior of the wood specimens was investigated with small burner and smoldering tests, as well as cone calorimeter (CC) and pyrolysis-combustion flow calorimetry (PCFC) measurements. The aim is to identify the starch variant that achieves the best flame retardancy compared to industrial flame retardant. Furthermore, it is discussed in which form the starch/urea/phosphate system should be applied on wood fiber. The simplest option is to use the starting components in unreacted form as physical mixture of biopolymer, phosphate agent, and urea. Another variant is the application of the uncleaned reaction products. They still contain greater amounts of unreacted educts and low molecular weight products. The use of the purified products represents the third possibility. The efforts and costs of providing the flame retardants increase in the specified order and are therefore part of the investigation. Furthermore, the industrial application capability of a starch-based FR was tested in a scale-up from an insulation manufacturer.

## 2. Results and Discussion

### 2.1. Synthesis

The synthesis in phosphate/urea systems is a common method to produce starch phosphates with a certain amount of nitrogen [19,23]. The powdery starting components are transformed by the melting of urea into a dough-like viscous structure that cannot be mixed by usual magnetic stirrer. In order to find out how the functionalization can be optimized, and to generate products with adjustable properties, experiments were carried out in the oven, kneader, and extruder. Whereas the oven synthesis is a static reaction, in the kneader and extruder processes the molten mass can be mixed during heating. The results of starch functionalization are summarized in Table 1. 

The synthesis in the oven leads to a phosphate content of 32.4 wt.% after only 30 min of reaction. This is a much shorter time compared to 2 h of reaction in the kneader with an additional mixing. The higher phosphate content after this short time can be attributed to the reaction under vacuum. The esterification of starch hydroxyl groups with phosphates is an equilibrium reaction. With the removal of water under vacuum, the balance can be shifted to the side of the products. The highest functionalization (38.8 wt.%) was achieved after 6 h of reaction in the kneader. However, even after 3 h, only slight changes in the phosphate level can be observed. The syntheses in the extruder demonstrate that the reaction method can be converted to a larger scale (sample *WS-E*). Urea phosphate (CO(NH_2_)_2_·H_3_PO_4_) was used as PA because it led to soluble products in preliminary experiments, which is important for industrial application. A brown colored product was obtained with a phosphate content of 16.1 wt.%. This is high in view of the short residence time of 2 min compared to the kneader. The thin rolling in the extruder ensures intensive mixing and high conversion rates. The total nitrogen content (N_tot_) remains low in all variants compared to the phosphate content and results mainly from the carbamation and the ammonium counterions. The comparison between total and ammonium nitrogen (NH_4_^+^) shows that only a small amount of carbamate nitrogen could be present overall, which is consistent with the results of other studies [23]. The solubility of products is important for the application process to the wood fibers, as well as for the protection effect. Short reaction times (in the cases of *WS-O*, *WS-K* after t_rct_ = 1 and *WS-E*) seem to favor the solubility of the products. Phosphates and carbamate groups can undergo crosslinking and condensation reactions under the release of water and ammonia, which could lead to the deterioration of solubility in the course of the running reaction. On the other hand, acidic phosphating agents (such as urea phosphate) and high mechanical stress, as in the extruder synthesis (sample *WS-E*), could lead to a degradation of the starch polymer chain and a better solubility. It may be assumed that the flame retardancy on the wood fiber is better if the additive is in dissolved form during application and completely covers the matrix material. Undissolved components are applied as dispersion. Thus, the additive would be present as small particles on the surface of the fiber with gaps in the covering.

### 2.2. Reaction to Fire Tests

Fire tests according to ISO 11925 have been used to assess the flammability of loose wood fibers with different additives exposed to a small flame (Table 2) [29]. This method is an established technique for the assessment and classification of the fire behavior of building materials according to DIN EN 13501 [30]. As references untreated wood fiber, native wheat starch (*WS*), as well as the commercial flame retardant *KF* and monoammonium phosphate (*MAP*) were compared. *KF* is an industrially used sulfur-based flame retardant of proprietary composition with low phosphate content. *MAP* was commonly used in the syntheses as phosphating agent. The flame-retardant effectiveness of the synthesized additives should therefore reach to at least the range of the pure *MAP*.

The wood fibers without additives and with the native starch achieved the worst results and reached the maximum fire cone height of 20 cm. Self-extinction after a total of one-minute test duration was not observed. *MAP* and the *KF* showed the best results with 9.1 and 10.7 cm. *KF* is not directly comparable to the phosphate-based biopolymers, but is used for the evaluation of the effectiveness of the flame retardants. The good result of the test specimen with *MAP* is due to the overall high phosphate content of the incorporated salt.

The starch-based additives were measured in three different variants of constitution:

- as physical mixture in the form of the unreacted components of the starting materials
- in the uncleaned form, after several hours of reaction
- in the form of the cleaned synthesis product

**Figure 3:**
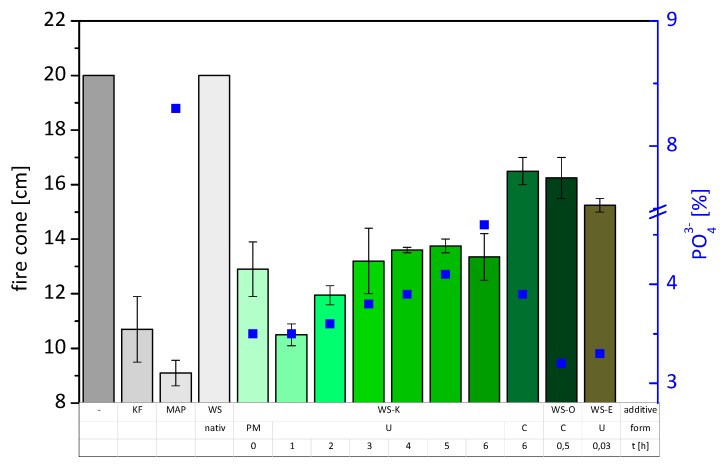
Results of fire test according to ISO 11925, fire cone height of the specimens with various additives, left to right: without additive, commercial flame retardant *KF*, pure *MAP*, native wheat starch (*WS*), several variations of *WS-K*, as physical mixture (*PM*), in uncleaned form (*U*), after different times of reaction, and in cleaned form (*C*) after 6 h reaction; phosphate content of the specimens ■.

From this comparison, further announcements should be made about to what extent a synthesis and purification of the products makes sense, because they are associated with energy and costs. Figure 3 illustrates the results of the measurement with regard to the phosphate content of the samples. Starch, urea and *MAP* as a physical mixture, provide protection up to 12.9 cm fire cone height (see Figure 3, *WS-K (PM)*). An improvement is achieved with the use of uncleaned *WS-K* after only one hour of reaction. But with increasing synthesis time, the performance subsides, although the phosphate content increases continuously. The fire cone height developed from 10.5 to 13.4 cm after 6 h of reaction. This effect may be attributed to the deteriorated solubility of the synthesis products. A good solubility leads to a better wetting of the fibers in the application process. Insoluble compounds were sprayed as a dispersion onto the fiber. It may be assumed that the FR efficiency is different, whether the FR lies on fiber surface as particles, or as complete cover. The solubility of the biopolymer compounds therefore plays a crucial role in the fire protection. The purified synthesis variant *WS-K (C)* shows the worst results after 6 h of reaction and exceeds the measurement mark of 15 cm according to the standard EN 13501-1. In this sample, well soluble by-products (mainly urea and *MAP*) were washed out, resulting in lower phosphate and nitrogen content. The remaining content was the poorly soluble starch derivative, which led to poor protection performance as discussed before. *WS-E* and *WS-O* had significantly worse test results despite good solubility. Note, however, that they have a lower phosphate and nitrogen content than the *WS-K* samples and are nevertheless in the range of *WS-K (C)*.

### 2.3. Smoldering Behavior

**Figure 4:**
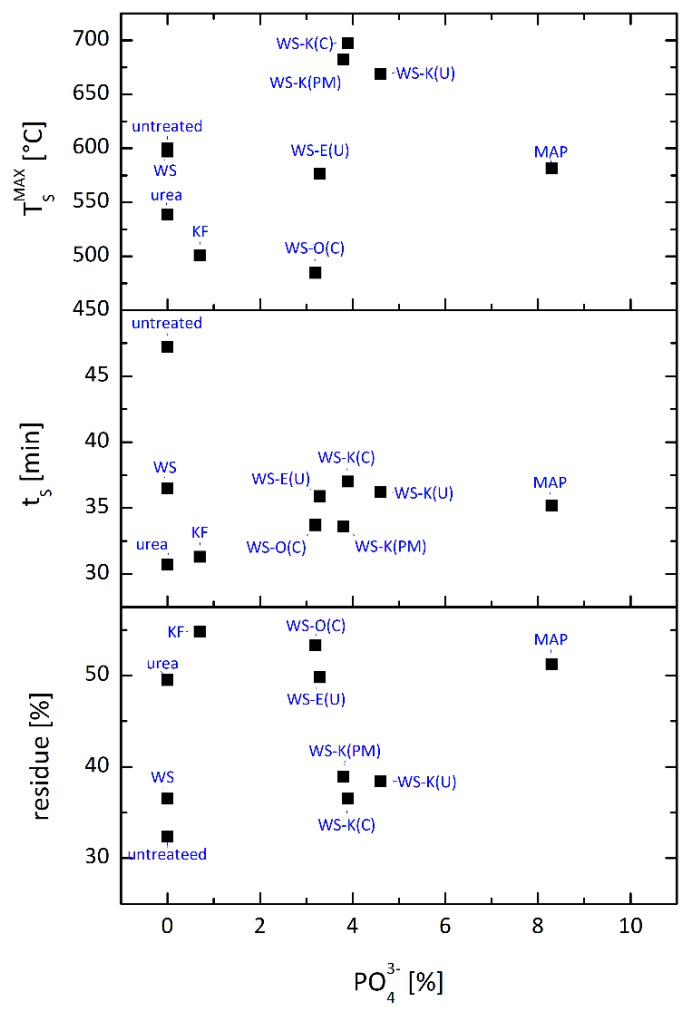
Residue, time (t_S_) and maximum temperature (T_S_^MAX^) from smoldering measurements of wood fibers with different additives depending on their phosphate content, additive content on fiber 10 wt.% (for detailed information see Table 3).

Smoldering is a slow oxidation of a solid fuel accompanied by temperature increase and smoke emission without open flame appearance. The phenomenon particularly occurs for porous organic materials like cellulose and wood. Smoldering is more difficult to suppress and detect than open fires, contributing to its dangerousness [31]. In rare cases, flame retardants also have effective protection against smoldering. Sometimes even flame retardants promote this process [32,33]. Therefore, the smoldering behavior of various additives was investigated by a method proposed by Hagen et al. [34]. The results are illustrated in Figure 4 and listed in Table 3.

**Figure 5:**
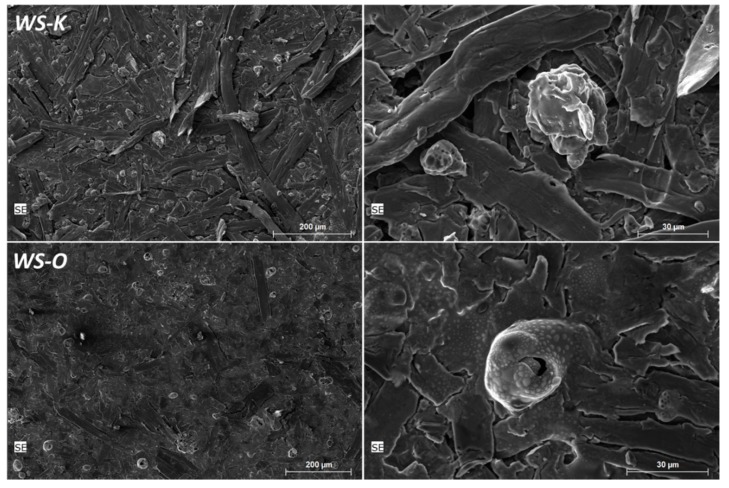
SEM images of wood fibers with 10 wt.% wheat starch-based FR *WS-K* (poorly soluble) and *WS-O* (well soluble) after heating to 350 °C in the oven (heating rate 10 K/min), recordings by Björn Günther using FEI Quanta ™ FEG 650.

The reference flame retardant *KF* (containing sulfur), as well as *MAP* significantly reduce the mass loss and smoldering time compared to wood fibers with native wheat starch and the untreated variant. This is consistent, as sulfur and phosphorus are known to reduce smoldering phenomena [35,36,37,38]. The addition of the starch-based FR to the fibers result in similar effects on residue and time. This is certainly also due to the contained phosphorus. It is noteworthy that the slightly soluble additives *WS-O* (*C*) and *WS-E* (*U*) has a significantly higher mass (53.3 and 49.8 wt.%) compared to the poorly soluble *WS-K* samples (36.5–38.9 wt.%). SEM images show the carbon-rich residues of the fibers treated with the poor (*WS-K*) and well (*WS-O*) soluble wheat starch after tempering to 350 °C (Figure 5). There are small fragments on the surface, probably from insoluble particles of *WS-K*. Thus, the protection is inhomogeneous and only partially given. In the close-up, small bubbles are visible in *WS-O* sample over large areas on the surface, indicating that the surface was covered better by the FR. *WS-O* and *WS-E* exhibit similar values in smoldering to *KF* and pure *MAP* which has a significantly higher phosphate content. The smoldering time is shorter for all starch FR samples than for untreated wood fiber. The higher temperatures of the *WS-K* samples compared to *MAP* is, finally, unclear. However, the application process of the FR additives could partially affect the pore structure of the fiber matrix during measurement. An adhesion of the fibers or minimal clumping by the additive entry cannot be excluded and could contribute to a modified oxygen supply of the combustion zone and a hotter burning.

### 2.4. Thermal Degradation

**Figure 6:**
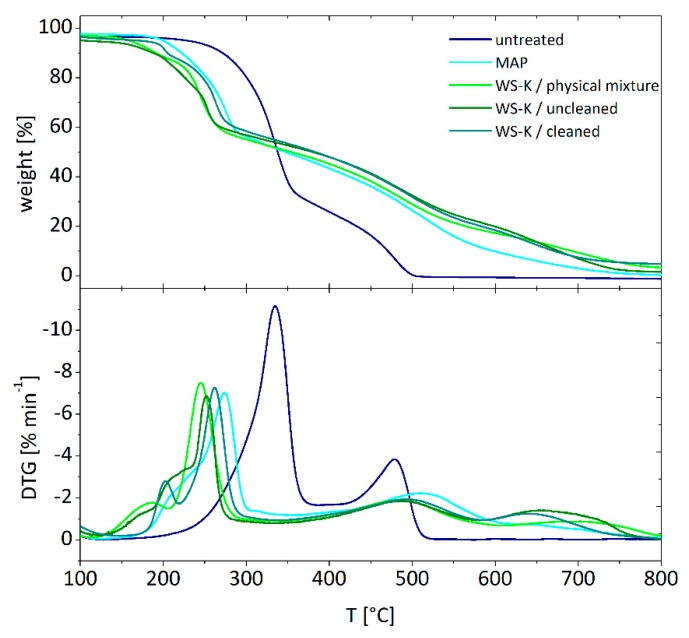
Thermal degradation behavior of wood fibers with different additives measured with TGA under air atmosphere, additive content 10 wt.%.

The thermal degradation behavior of starch-based FR on wood fiber was investigated by thermogravimetric analysis (TGA). The major weight loss of untreated wood takes place in two steps at maximum weight loss rate temperatures (T_Peak_) of 335 and 479 °C (see Figure 6 and Table 4). 

In accordance with the literature, the first stage can be attributed mainly to the decomposition of the polysaccharide’s cellulose and hemicellulose. Hemicellulose has an amorphous structure and is composed of different monomer building blocks (such as xylose, mannose, glucose, etc.) with short side chains and branches. They decompose even at low temperatures to volatile components such as CO, CO_2_ and low molecular weight hydrocarbons [39]. Cellulose is a semi-crystalline polymer and consists of unbranched chains of glucose molecules [40]. This results in a higher stability against thermal stress compared to hemicellulose. In accordance with the literature, the decompositions of hemicellulose and cellulose overlap and form a single decomposition step [41]. Lignin, the third main component of wood, has a complex 3-dimensional structure and its aromatic building blocks are linked by different types of bonding [42]. Therefore, lignin is thermally more stable. It decomposes, depending on the origin and composition, under pyrolytic conditions in a wide temperature range up to 900 °C [39,43,44]. Therefore, the second phase at 479 °C could be attributed in part to the degradation of lignin. In addition, secondary solid phase reactions could take place through the oxidation of carbonaceous residues from previous degradation processes [45]. Since the degradation of wood is accelerated by oxygen, the decomposition of the untreated wood material is nearly completed at about 500 °C.

The addition of the phosphate-containing additives (*MAP*, *WS-K*) causes a shift of the first degradation phase to lower temperatures and to higher temperatures of the second phase. It is known that phosphorus-containing flame retardants are able to act as an acid catalyst in the condensed phase, causing charring by esterification and dehydration in temperature ranges prior to the decomposition of the original materials [46]. Furthermore, the incorporation of phosphorus improves the thermo-oxidative stability of the char at high temperatures [47].

The shoulder of the peak at 274 °C may occur due to the volatilization of ammonia [48]. *MAP* decomposes at approximately 210 °C to the following: NH_4_H_2_PO_4_ → H_3_PO_4_ + NH_3_↑

This decomposition can also be assumed in sample *WS-K (U)*, since it shows a shoulder in the same range and still contains free monoammonium phosphate from the synthesis. However, the first degradation phase of all *WS-K* variants is preceded by a smaller weight loss, which appears as a resolved peak in *WS-K (PM)* and *WS-K (C)* at 187 and 201 °C and as a two-step shoulder in *WS-K (U).* The first step of the shoulder from *WS-K (U),* and the small peak at 187 °C could have been caused by urea, which starts to decompose as it melts at 133 °C into volatile ammonia and isocyanic acid (see Figure 1) [49]. In variant *WS-K (C)* urea was washed out, the weight loss no longer observable. The peak at T_Peak_ = 201 °C of the purified sample *WS-K* also occurs as part of the shoulder in variant *WS-K (U)*. In comparison with DTG curves of the pure *WS-K (C)* additive (i.e., not applied on wood fiber), the peak can be assigned to the decomposition of the starch derivate itself (not shown in Figure 6, see Table 4 or DTG curve in Supplementary Materials). In the further course of the TG measurements of the *WS-K* samples, there are only slight differences. All three variants, however, show new degradation phases at 639, 654, and 698 °C compared to *MAP* and the untreated wood fiber. The evaluation of the DTG curve from *WS-K (C),* measured in pure form (see Supplementary Materials), suggests again that the step results from the oxidation of the carbon-rich residues of the starch degradation products. However, the peaks differ slightly in temperature, shape, and intensity. The additive form (physical mixture, uncleaned, cleaned syntheses variant) therefore has a considerable influence on the char. Overall the *WS-K* samples show a significantly higher residual mass (29.0–31.9 wt.%) at 500 °C than the untreated wood fiber (0.5 wt.%) and a slightly higher than pure *MAP* (26.5 wt.%), although the phosphate content on the wood fiber is about twice as high. Since the composition of the commercial flame retardant *KF* is unknown, the degradation behavior cannot be discussed in detail. It is listed to evaluate the stability of the decomposition products with an industrial flame retardant. Thus, the residual mass of the *WS-K* samples is approximately twice as high at 500 °C as that of the *KF* sample.

### 2.5. Cone Calorimeter Measurements

**Figure 7:**
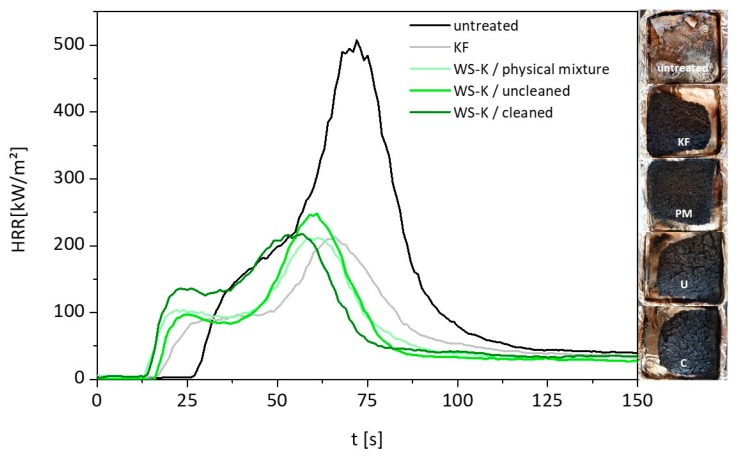
Left, heat release rate curves from CC measurement of untreated wood fibers without additive (reference), with commercial flame retardant *KF* and starch-based *WS-K* in form of physical mixture (*PM*), uncleaned (*U*) and cleaned synthesis product (*C*); right, photos of the residues from the cone calorimeter tests.

The results of the cone calorimeter (CC) measurements are illustrated in Figure 7 and summarized in Table 5. Unmodified wood fibers exhibit the highest peak of heat release rate (pHRR) of almost 500 kW/m^2^. The curve follows the typical shape of a non-charring sample [50]. Ignition occurs at 31 s. The HRR curve increases fast to a slight shoulder. After this shoulder, HRR rises to its maximum at about 75 s. There are two ways to explain this curve shape. Either the surface starts to break up at this point and oxygen penetrates into the porous sample so that the wood fiber starts to burn more efficient [51], or the heat front reaches the bottom of the sample and cannot be removed due to insulation condition. Therefore, temperature and pyrolysis rate increase. Overlapping effects are also possible. After pHRR, the rate decreases quickly up to around 80 kW/m^2^ and then becomes much slower. This last step corresponds to the smoldering process due to the oxidation of the residue when the flame is vanishing. The sample residue at the end is negligible. The starch and commercial FR additive exhibit a much lower pHRR (209–233 kW/m^2^), TTI (14–18 s), THR (around 10 kJ/g) and a higher residue content (17–24 wt.%). Compared to the unmodified wood fiber, the HRR profile looks different. The shoulder has changed to a small peak followed by a plateau. The plateau corresponds to the formation of a protective char layer, where oxygen and heat cannot penetrate so fast inside the sample [52].

The assessment of smoke release is an important criterion because black smoke restricts visibility, making it difficult for occupants to escape [53]. All treated wood fibers show a lower total smoke production (TSP, calculated from the generated smoke divided by the initial sample weight) in comparison to the untreated one. The suppression of smoke formation is typical for flame retardants that work in the condensed phase.

The residue at the end of the test is much more stable, which is common for materials with phosphorus-based FR [54]. Consequently, carbon rich fibers are still observed, and the residue remains black (see Figure 7, right side).

The differences in TTI compared to unmodified reference can be assigned to the lower thermal stability of modified wood fibers, as expected for phosphorus-modified materials, and as confirmed by TG and PCFC analyses. Overall, there are few differences between starch based and commercial FR samples. A clear trend between physical mixture, cleaned and uncleaned synthesis product from starch is not apparent. The effective heat of combustion (EHC) corresponds to the combustion of volatiles from the material. It is calculated from the ratio of total heat evolved and mass loss within a specified time [55]. EHC for unmodified wood is slightly higher as normal and may be due to the glue used to bind the fibers [56]. EHC of modified fibers decreases comparing to unmodified ones. This is consistent because the use of P and N containing flame retardants results in release of water and nitrogen compounds that cool and dilute the combustion gases. Moreover, a higher char content entraps high amount of carbon contributing to decrease the heat released by gas combustion.

### 2.6. Pyrolysis Combustion Flow Calorimeter Measurements

**Figure 8:**
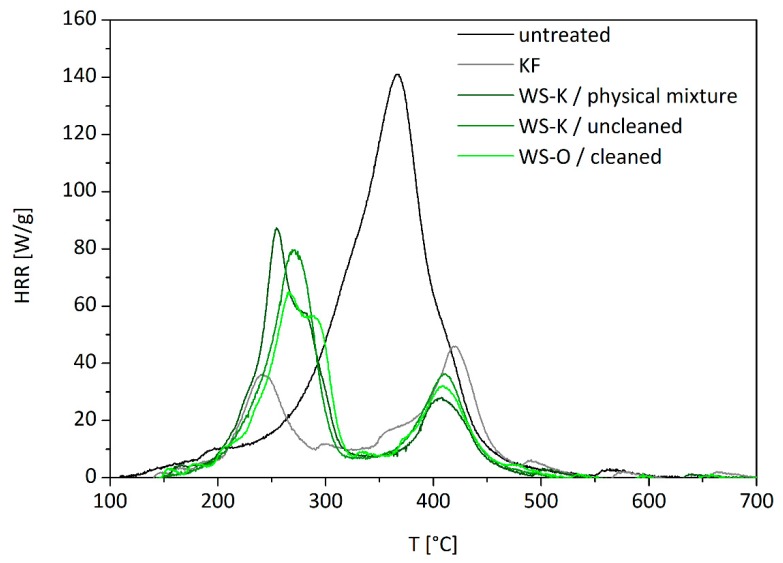
Heat release rate curves from PCFC measurements of untreated wood fibers without additive, with commercial flame retardant *KF* and starch-based FR *WS-K* and *WS-O* in form of physical mixture, uncleaned and cleaned synthesis product.

Pyrolysis combustion flow calorimeter (PCFC) was used to characterize the flammability and thermal decomposition of biopolymer-based FR on wood fiber. The experimental data are shown in Figure 8 and summarized in Table 6.

Unmodified wood fibers exhibit a pHRR of 128 W/g at 360 °C, a total heat release rate (THR) of 13.3 kJ/g, a residue content of 13 wt.%, and a heat of complete combustion of 15.3 kJ/g. These values are typical for cellulose-based materials (even if heat of combustion is slightly higher than expected as already mentioned in the section above) and quite low compared to many synthetic polymers [57]. In contrast, all flame retarded samples exhibit a two-step decomposition. The first peak is the highest at low temperature (240–300 °C). This decrease in thermal stability is due to the presence of acid dehydration substance like phosphates. The reduction in THR is caused by the char promotion effect of the FR (from 13.5 to 34.0 wt.%), but also to a decrease of heat of combustion. Indeed, the residue is enriched in carbon and the combustion enthalpy stored in the char is generally high. The second pHRR is observed at higher temperature with low intensity (400–420 °C). This peak is usually rarely observed in cellulosic biomass. While wood is richer in lignin, it may be due to the decomposition of lignin [58,59]. There is no great difference between the HRR of starch-based FR except for the cleaned variant of *WS-K* which exhibits worse performances with high pHRR, high THR, and the lowest residue from all FR samples. In contrast, the cleaned *WS-O* variant shows significantly better performance, although it has a lower phosphate content. A possible explanation is that the better solubility of the *WS-O* sample leads to a better covering of the fibers as discussed earlier.

In contrast to the cone calorimeter measurements, the solid state and the gas phase combustion are separated in the PCFC. Thermo-oxidation occurs in cone calorimeter at the end of the test. To compare effective heat of combustion in CC with heat of complete combustion in PCFC, the effective heat of combustion (EHC) from HRR was calculated when HRR stabilized at about 80 kW/m^2^ and thermo-oxidation starts. In that case, EHC corresponds mainly to combustion of fuels released by anaerobic pyrolysis. From the ratio of EHC and HCC (from PCFC) the combustion efficiency χ can be calculated approximatively (see Table 7). Its value is close to 1 in all cases, confirming that there is no flame inhibition in the gas phase and the charring effect in the condensed phase is predominating, as it is expected for phosphate FRs.

### 2.7. Industrial Application

**Figure 9:**
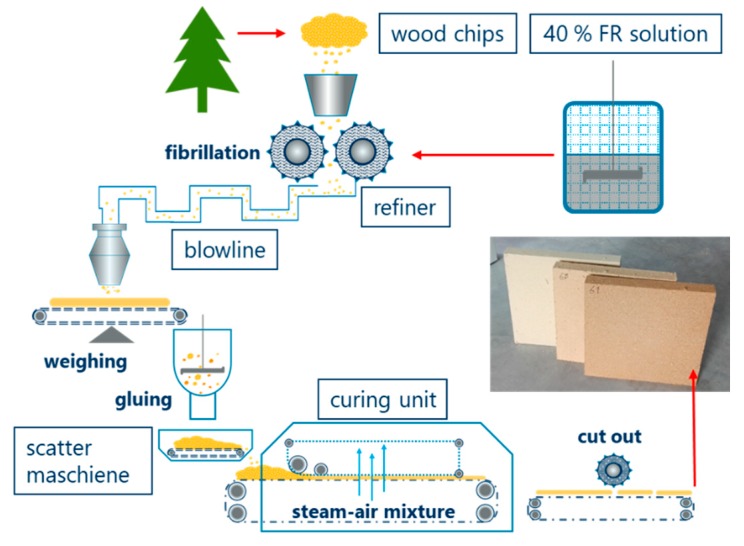
Scheme of the production line for the application of starch-based flame retardants to wood fiber material (illustration adapted from [61]), and photo of the wood fiber products (from left to right) without, with 5 and 10 wt.% fire retardant additive *WS-E*.

The industrial applicability of starch-based flame-retardant (*WS-E*) has been proven on wood fiber material in the production plant of an insulation manufacturer (GUTEX, Waldshut-Tiengen, Germany). The processing took place in the so-called dry-process involving the following steps: defibering of the wood chips by a refiner, fiber drying to the residue moisture necessary for the gluing process, gluing with PMDI, scattering of the fibers on the forming belt to a mat, curing the mat with steam air mixture, cutting to size, and packaging [60]. The production scheme is illustrated in Figure 9.

**Figure 10:**
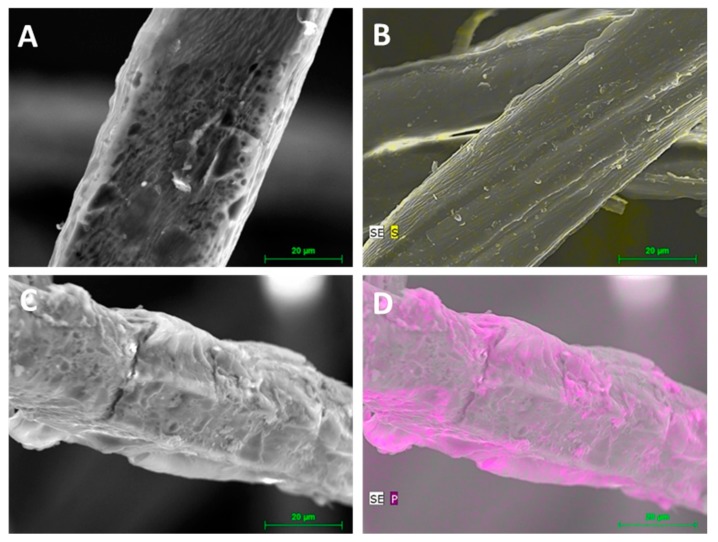
SEM images of untreated wood fibers (**A**), with 10 wt.% commercial flame retardant *KF* (**B**), with 10 wt.% starch-based flame retardant *WS-E* (**C**,**D**), visualization of the element distribution S and P by EDX in yellow and violet (**B**,**D**), recordings by Björn Günther using JEOL JSM-T330A.

The basic requirement for the application was a good solubility, a property that determines whether the FR can be integrated into existing dosing systems and how much water is needed to incorporate it into the product matrix. The choice was therefore the well-soluble starch derivative *E-WS* (solubility 95 wt.%). Moreover, we have previously shown that solubility is favorable to reach a high flame-retardant efficiency. Analogous to the conventionally used commercial FR additive *KF*, the powdery *E-WS* was stirred in a 1000 L intermediate bulk container to a 40 wt.% aqueous solution. Meanwhile a slight foaming could be observed by the stirring, but a clumping of the FR did not occur. The additive solution was added directly to the refiner process to achieve a homogeneous distribution. In total, about 500 kg of flame retardants were processed. A photo of the finished wood fiber board is included in Figure 9. SEM images show the application of the commercially used flame retardant *KF* and the starch-based *E-WS* compared to untreated wood fiber (Figure 10). 

The images using energy dispersive X-ray spectroscopy (EDX) document a very homogeneous distribution of the sulfur containing industrial FR on the fiber. However, the starch FR lies on the fiber, enclosing it like a shell. The visualization by EDX shows areas with more intense color, which indicates a partially higher phosphorus concentration and less homogeneity. The recovery was determined by measuring the phosphorus content of the produced insulating material divided by the expected phosphorus content after the application of the FR on the wood fiber multiplied by hundred. The recovery rate was only 53 ± 0.05 wt.% with generally good reproducibility. The losses of flame retardants can have several causes. Losses due to rinsing and sticking to pipes and vessel walls occur, but are too low to explain losses of 47 wt.%. The starch-based FR has a slightly higher viscosity compared to the otherwise used industrial FR. The dosing unit could therefore have worked imprecisely. For further experiments, an adjustment of the dosage would be appropriate.

## 3. Experimental Part

### 3.1. Materials

All starting polymers used for the flame-retardant synthesis are commercially available and were used without further pretreatment. Wheat starch was purchased from Jäckering Mühlen- und Nährmittelwerke GmbH (Hamm, Germany). The commercial reference flame retardant *KF* is a sulfur-based product and was provided by GUTEX Holzplattenfaserwerk H. Henselmann GmbH & Co. KG (Waldshut-Tiengen, Germany). Urea and phosphate agents were obtained from Carl Roth GmbH + Co. KG. Loose untreated softwood fibers from GUTEX were used as matrix material for the fire behavior characterization. The additives were applied to the fiber material in different forms: in cleaned form (*C*, i.e., the synthesis products were dialyzed to remove unreacted urea and phosphating agent), in uncleaned form (*U*, unreacted PA and urea are included), and as physical mixture (*PM*, i.e., no synthesis took place, the composition corresponds to the synthesis mixture at t_rct_ = 0).

### 3.2. Syntheses

The syntheses were carried out as described by Heinze et al. in a laboratory oven, kneader, and extruder at the parameters given in Table 1 [19]. The achieved phosphate and nitrogen content of the products depend on the synthesis variant (oven, kneader, extruder) and the reaction time (t_rct_), and is given in the *Results and Discussion* section. The purification of the products was carried out by dialysis (membrane: SpectraPor^®^ 3, MWCO: 3500 Da, Spectrum Labs) against deionized water until the conductivity decreased constancy below 10 μS.

#### 3.2.1. Oven Synthesis

The synthesis was carried out in a vacuum drying oven. For this purpose, the starch/PA/urea starting mixture was distributed on a stainless steel tray, homogenized with water and predried at 60 °C. The reactions take place at 140 °C and 0.07 bar for 0.5 h in a molar ratio of AGU_starch_/MAP/urea from 1:3:6.

#### 3.2.2. Kneader Synthesis

The synthesis was carried out on a laboratory universal mixing and kneading machine LUK 2.5 (Werner & Pfleiderer GmbH Maschinenfabrik, Stuttgart, Germany) with two crank-shaped moving Z-kneading blades. The starting materials for the synthesis were added without previous homogenization in powder form in the kneading trough. The functionalization took place in a molar ratio of AGU_starch_/MAP/urea from 1:3:4, a kneading arm rotation speed of 45 rpm and a jacket temperature of 160 °C.

#### 3.2.3. Extruder Synthesis

The scale-up was done by Jäckering Mühlen- und Nährmittelwerke GmbH in the technical center Bochum of the company ENTEX Rust & Mitschke GmbH with a planetary roller extruder. A mixture of wheat starch and urea phosphate in a molar ratio of 1:1 was used. The technical data of the system are given in Table 8.

### 3.3. Methods

#### 3.3.1. Laboratory-Scale Application

The flame-retardant application was carried out on loose wood fibers from GUTEX with an additive content of 10 wt.%. The additives were applied to the fibers by spraying in the form of a 10 wt.% aqueous solution or dispersion. Slightly soluble compounds were finely dispersed prior to spraying with a high-performance dispersing machine (Batch-Ultraturrax T 25 basic, IKA^®^ Werke GmbH & Co. KG, Staufen, Germany). The fibers were air dried in a fume hood to a dry content of 94 wt.%.

#### 3.3.2. Industrial Application

The starch-based FR from the extruder synthesis (*WS-E*) was tested by GUTEX for the application in the industrial production of wood fiber-based insulating materials. The FR was added during defibration of the wood chips in the refiner process in form of a 40 wt.% aqueous solution. The dosage was chosen so that insulation with 5 and 10 wt.% FR content should be produced.

#### 3.3.3. Characterization

Phosphorus contents were analyzed by inductive coupled plasma spectroscopy ICP-OES CIROS CCD from SPECTRO Analytical Instruments at the Institute of Soil Sciences and Site Ecology (TU Dresden). Prior to ICP measurements, the dry samples were subjected to microwave assisted digestion based on DIN EN 13805:2002 [62]. The values were converted and are given in the text as phosphate contents. The degree of substitution of phosphate groups (DS_P_) was calculated using the following equation:

(1) $${\mathrm{DS}}_{P}=\frac{\frac{\left[{\mathrm{PO}}_{4}^{3-}\right]}{100}\cdot 162}{95\left(1-\frac{\left[{\mathrm{PO}}_{4}^{3-}\right]}{100}\right)}$$

with AGU being the anhydroglucose unit, [PO_4_^3−^] the determined percentage of phosphates via ICP, 95 the molar mass of phosphates and 162 the molar mass of the AGU of starch. Total nitrogen contents were conducted using the device vario EL III from Elementar Analysensysteme GmbH, Langenselbold, Germany. The determination of the ammonium nitrogen was carried out with a *Parnas-Wagner* apparatus as descripted in [23]. Scanning electron microscope (SEM) images were made by Björn Günther at the Institute of Forest Utilization and Forest Technology with a JEOL JSM-T330A (JEOL Technics Ltd., Tokyo, Japan) equipped with an EDR Röntec M5 and energy dispersive X-ray spectroscopy and a FEI Quanta™ FEG 650. The evaluation was done with the software Quantax 400 from Bruker, AXS Microanalysis GmbH. Thermogravimetric investigations were carried out on a Netzsch STA 449 F5 Jupiter^®^ from NETZSCH-Gerätebau GmbH (Selb, Germany). For the measurements, 5–10 mg of sample material were weighed into alumina crucibles. The samples were heated to 900 °C at a heating rate of 10 K/min under synthetic air atmosphere. The evaluation and generation of the DTG curves was carried out with the Netzsch Proteus Thermal Analysis software.

The water solubility of the products was determined gravimetrically at room temperature. Therefore, 0.2–0.4 g of ground sample (m_s_) were stored in 40 mL of water for 24 h to ensure a complete dissolution. The water-soluble fraction was separated by centrifugation and the insoluble residue was washed twice with deionized water. The solutions were collected and dried at 105 °C to constant mass (m_ws_). The calculation is done according to Equation (2):

(2) $$\mathrm{solubility}=\frac{m_{\mathrm{WS}}}{m_{S}}\times 100\%$$

**Figure 11:**
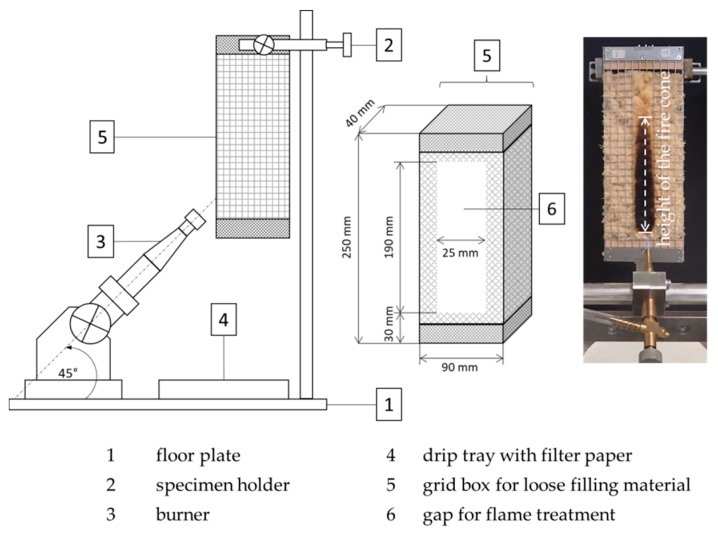
Schematic illustration of the fire test according to ISO 11925-2 and exemplary photo of a specimen after flaming [29].

The flammability was tested on wood fibers in a small burner test based on ISO 11925-2 [29]. The experimental setup is outlined in Figure 11. The fiber material was filled in wire mesh boxes with a fiber density of 30 kg/m^3^. The samples were ignited for 15 s. The assessment of the flammability and flame propagation took place on the height of the fire cone, which was determined after 60 s test duration by measuring the span of the charred sample surface.

**Figure 12:**
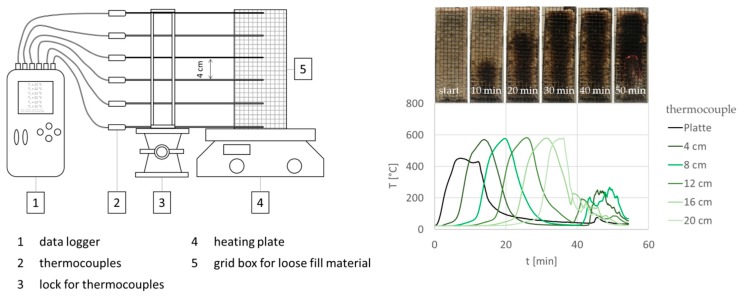
**Left**, Scheme of the experimental setup for smoldering measurements of loose wood fiber; **right**, exemplary temperature curves with photos of the fibers as a function of smoldering time.

The smoldering behavior was investigated according to a test proposed by Hagen et al. (experimental setup see Figure 12) [34]. The fibrous material was filled in upwardly opened wire mesh boxes (200 × 65 × 65 mm) with a target density of 30 kg/m^3^. The data were recorded with a logger OM-DAQPRO-5300 of the company OMEGA Engineering GmbH Deckenpfronn, Germany with type K thermocouples (probe length 200 mm, probe width 1.5 mm) of the company JUMO GmbH & Co. KG Fulda, Germany. To monitor the smoldering temperature curve and determine the maximum temperature (T_S_^MAX^), 6 thermocouples were fixed at distances of 4 cm each in the center of the grid box. The ignition source was a heating plate of the type Yellow MAQ HS from IKA^®^-Werke GmbH & CO. KG, Staufen Germany. After reaching the ignition temperature (350 °C, after 5 min), the hot plate was switched off and the smoldering process spread independently. The residue was determined gravimetrically from the mass ratio of the sample material after the smoldering process finished and the starting mass multiplied by hundred. The smoldering time (t_S_) is defined as the duration from the start of the heating phase until the end of the process, when all probes indicate a temperature below 100 °C. All samples were tested in duplicate.

Calorimetric measurements were executed at the Centre des Materiaux des Mines d’Alès (C2MA), Ecole des Mines d’Alès, in France. Pyrolysis-combustion flow calorimetry (PCFC) was carried out under anaerobic pyrolysis with heating rate of 1 K/s up to 750 °C and combustion in excess of oxygen at 900 °C (method A according to ASTM D 7309). The cone calorimeter experiments were done using a spark igniter and with a heat flux of 35 kW/m^2^ according to ISO 5660 standard. The dimensions of the specimens were 100 × 100 × 3 mm^3^ at a density of 500 kg/m^3^. The binder used for the fibers was Acrodur DS 3558 from BASF. The binder was sprayed onto the fibers in a proportion of 10 wt.% in a gluing aggregate from DRAIS. All samples were tested in duplicate.

## 4. Conclusions and Outlook

Flame retardants based on wheat starch modified with phosphate/urea systems were tested for the first time as additives for wood fiber material. Their flame-retardant properties were extensively characterized with small burner and smoldering tests, thermogravimetric analysis, and cone calorimeter, as well as pyrolysis combustion flow calorimeter. Flame-retardant efficiency depends on the phosphate content, solubility, reaction time, and the additive form (physical mixture, uncleaned, or cleaned synthesis products). The application of well-soluble additives has a positive effect on fire and smoldering protection. The calorimetric measurements show a significant reduction in heat release rate compared to untreated wood fibers. It could be confirmed that the flame retardants act exclusively in the condensed phase. In further studies, the transfer of the PA/urea reaction system to other biopolymers such as hemicellulose, lignin, and proteins would be interesting to achieve even better flame retardancy effects. The applicability of the additives on wood fiber was demonstrated by an industrial scale-up. The investigations prove that starch-based flame retardants are suitable, representing an alternative to common flame retardants in the wood fiber industry.

## Figures and Tables

**Table 1:** Reaction conditions and results of wheat starch functionalization in PA/urea systems.

Sample	Variant	Molar Ratio	PA Type	t_rct_	T_rct_	PO_4_^3−^	DS_P_	N_tot_	NH_4_^+^	Solubility
		AGU_starch_/PA/urea			[°C]	[wt.%]		[wt.%]	[wt.%]	[wt.%]
*WS-O*	Oven *	1:3:6	NH_4_H_2_PO_4_	0.5 h	140	32.4	0.8	6.7	6.1	82.7
*WS-K*	Kneader	1:3:4	NH_4_H_2_PO_4_	1 h	160	14.8	0.3	2.7	2.2	61.1
				2 h		29.4	0.7	5.8	5.4	01.4
				3 h		37.4	1.0	7.9	7.0	02.1
				4 h		36.7	1.0	7.6	6.8	02.3
				5 h		37.7	1.0	7.8	7.0	01.8
				6 h		38.8	1.1	8.3	7.0	02.1
*WS-E*	Extruder	1:1:0	CO(NH_2_)_2_·H_3_PO_4_	2 min	≈165	16.1	0.33	2.9	1.7	95.0

AGU anhydroglucose unit, PA phosphating agent, t_rct_ reaction time, T_rct_ reaction temperature, PO_4_^3−^ phosphate content, N_tot_ total nitrogen content, NH_4_^+^ ammonium ion content, DS_P_ degree of substitution of phosphate groups, * syntheses under vacuum (*p* = 0.07 bar).

**Table 2:** Results of the fire tests based on ISO 11925, additive content on the wood fiber 10 wt.%.

Additive	t_rct_	Form	Fire Cone Height	Solubility	PO_4_^3−^	N_tot_
	[h]		[cm]	[wt.%]	[wt.%]	[wt.%]
*References*						
untreated			20.0		0.0	0.0
*KF*			10.7	99.0	0.7	2.1
*MAP*			09.1	99.9	8.3	1.7
*WS*		native	20.0	01.3	0.0	0.1
*starch based*						
*WS-K*	0.00	PM	12.9		3.5	2.0
	1.00	U	10.5	61.1 ^a^	3.5	2.0
	2.00	U	12.0	01.4 ^a^	3.6	1.9
	3.00	U	13.2	02.1 ^a^	3.8	1.9
	4.00	U	13.6	02.3 ^a^	3.9	1.7
	5.00	U	13.8	01.8 ^a^	4.1	1.6
	6.00	U	13.4	02.1 ^a^	4.6	1.6
	6.00	C	16.5	02.1 ^a^	3.9	0.8
*WS-O*	0.50	C	16.3	82.7 ^a^	3.2	0.7
*WS-E*	0.03	U	15.3	95.0 ^a^	3.3	0.9

^a^ solubility of the cleaned product, t_rct_ reaction time, PM physical mixture, U uncleaned, C cleaned, PO_4_^3−^ and N_tot_ based on content in the test specimen.

**Table 3:** Results of the smoldering tests.

Additive	Form	PO_4_^3−^ [wt.%]	Residue [wt.%]	t_S_ [min]	T_S_^MAX^ [°C]
*References*					
untreated		0.0	32.4	47.2	597
*MAP*		8.3	51.2	35.2	581
*urea*		0.0	49.5	30.7	539
*KF*		0.7	54.8	31.3	501
*wheat starch based*					
*WS*	Native	0.0	36.5	36.5	600
*WS-K*	PM	3.5	38.9	33.6	682
*WS-K*	U	3.8	38.4	36.2	669
*WS-K*	C	3.9	36.5	37.0	697
*WS-O*	C	3.2	53.3	33.7	484
*WS-E*	U	3.3	49.8	35.9	577

Additive content on wood fiber 10 wt.%, PM physical mixture, U uncleaned, C cleaned, PO_4_^3−^ based on content of the test specimen, t_S_ smoldering time, T_S_^MAX^ maximum smoldering temperature.

**Table 4:** Results from TGA measurements.

Sample	Additive	PO_4_^3−^	T_Peak_	m_500°C_
		[wt.%]	[°C]	[wt.%]
*WS-K (C)*	-	38.8	202, 263, 493, 649	52.2
wood fiber	untreated	00.0	335, 479	00.5
	*KF*	00.7	239, 321, 497	15.1
	*MAP*	08.3	140–245(sh), 274, 515	26.5
	*WS-K (PM)*	03.5	187, 246, 488, 698	29.0
	*WS-K (U)*	04.6	130–230(sh), 251, 487, 654	32.5
	*WS-K (C)*	03.9	201, 262, 494, 639	31.9

Additive content on wood fiber 10 wt.%, T_Peak_ temperature of maximum weight loss rates from DTG curves, m_500 °C_ residual mass at 500 °C, *KF* commercial flame retardant, sh shoulder.

**Table 5:** Results from cone calorimeter measurements, test matrix wood fiber, additive content 10 wt.%.

Additive	Form	TTI	pHRR	THR	Residue	EHC	TSP	PO_4_^3−^	N_tot_
		[s]	[kW/m^2^]	[kJ/g]	[wt.%]	[kJ/g]	[m^2^/g]	[wt.%]	[wt.%]
*References*									
untreated		31	486	15.9	02.5	16.3	0,097		
*KF*		19	209	10.3	22.9	13.3	0.029	0.7	2.1
*wheat starch-based*									
*WS-K*	PM	14	224	10.6	18.3	13.0	0.030	3.5	2.0
*WS-K*	U	16	242	09.8	17.5	11.9	0.037	4.6	1.6
*WS-K*	C	16	244	11.4	17.4	13.8	0.063	3.9	0.8
*WS-O*	C	17	235	10.1	24.4	13.3	0.041	3.2	0.7
*WS-E*	U	18	243	10.6	21.4	13.5	0.051	3.3	0.9

TTI time to ignition, pHRR peak heat release, THR total heat release, EHC effective heat of combustion, TSP total smoke production, *KF* commercial flame retardant, PM physical mixture, U uncleaned, C cleaned.

**Table 6:** Results from the pyrolysis-combustion flow calorimetry, test matrix wood fiber, additive content 10 wt.%.

Additive	Form	T_1_	p_1_HRR	T_2_	p_2_HRR	THR	Residue	HCC	PO_4_^3−^	N_tot_
		[°C]	[W/g]	[°C]	[W/g]	[kJ/g]	[wt.%]	[kJ/g]	[wt.%]	[wt.%]
*References*										
untreated		363	128	-	-	13.3	13.5	15.3	0.0	0.0
*KF*		241	35	418	40	05.7	30.2	08.1	0.7	2.1
*wheat starch-based*										
*WS-K*	PM	270	74	400	26	06.4	30.7	09.1	3.5	2.0
*WS-K*	U	273	77	411	33	06.4	29.8	09.0	4.6	1.6
*WS-K*	C	299	116	417	39	09.4	23.6	12.2	3.9	0.8
*WS-O*	C	267	68	412	31	06.5	32.9	09.6	3.2	0.7
*WS-E*	U	282	94	416	33	08.0	28.8	11.2	3.3	0.3

t_x_ time to Peak, p_X_HRR heat release rate at peak X, THR total heat release, HCC heat of complete combustion, PM physical mixture, U uncleaned, C cleaned, *KF* commercial FR.

**Table 7:** Determination of combustion efficiency χ from CC and PCFC data.

Sample	EHC_CC_	HCC_PCFC_	χ
	[kJ/g]	[kJ/g]	
*KF*	10.0	8.1	1.2
*WS-K (PM)*	9.8	9.1	1.1
*WS-K (U)*	9.7	9.0	1.1
*WS-K (C)*	11.3	12.2	0.9
*WS-O (C)*	9.6	9.6	1.0
*WS-E (U)*	10.6	11.2	0.9

EHC_CC_ effective heat of combustion, HCC_PCFC_ heat of complete combustion, χ combustion efficiency, PM physical mixture, U uncleaned, C cleaned.

**Table 8:** Technical specifications of the extruder system used for the FR synthesis.

Component	Description
Manufacturing company	ENTEX Rust & Mitschke GmbH
Type	TP WE 70/1600 M4
Dosing unit	DDW-SR-20
Drive power	46 kW
Maximum spindle torque	2000 Nm
Maximum rotational speed	250 min^−1^
Number of modules	4
Number of side-feeder	4
Number & type of planetary spindles	5/standard, continuously interlocked
Gap dimension of dosing rings	3 mm

## Supplementary Materials

The following are available online, Figure S1: TG and DTG curve from *WS-K (C)* in pure form.
