# Supplementary figures and images for: Flame Retardancy of Wood Fiber Materials Using Phosphorus-Modified Wheat Starch
Source: Molecules. 2020 Jan 14;25(2):335. doi: 10.3390/molecules25020335 (PMC7024314; doi:10.3390/molecules25020335)

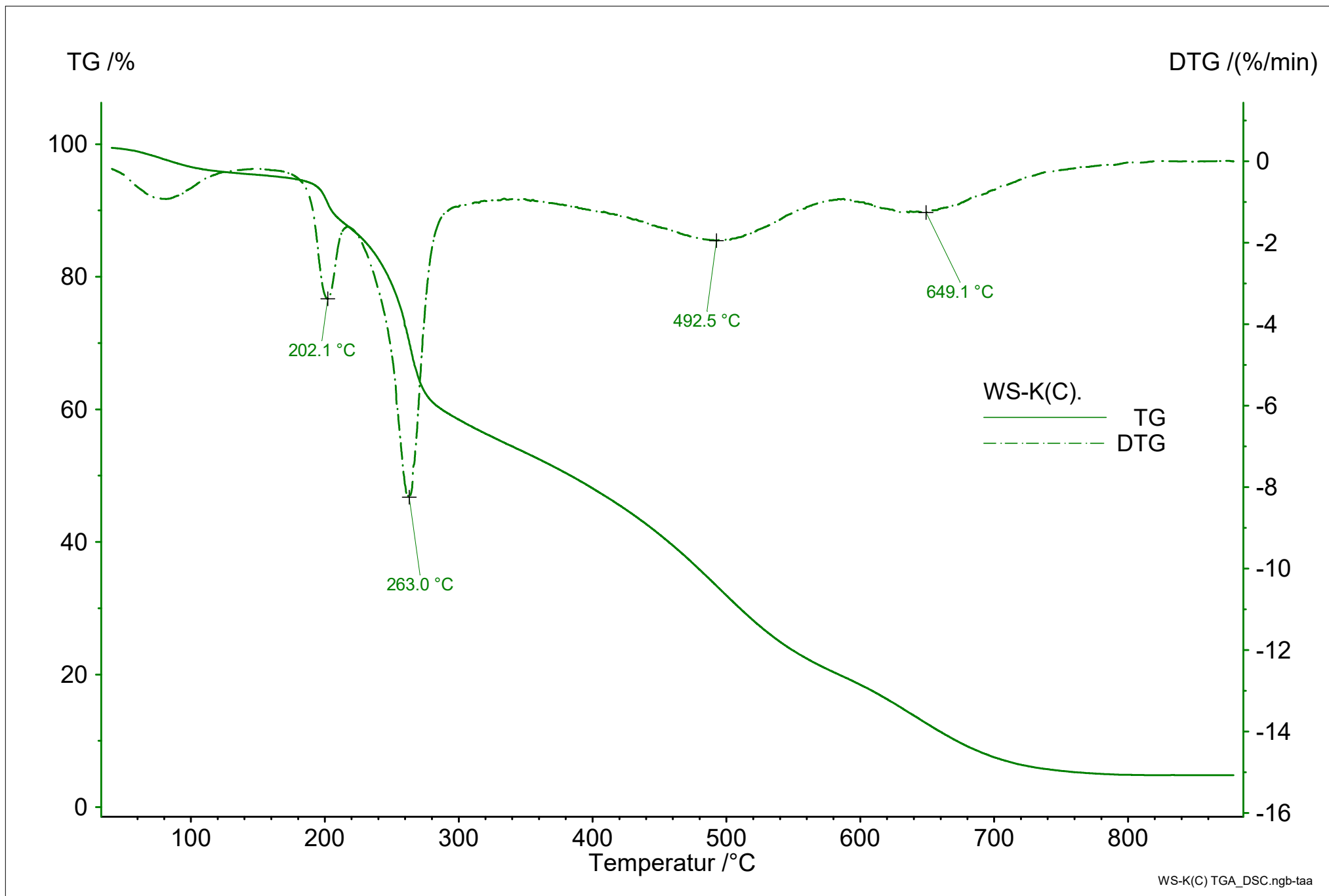

Supplement: Supplementary file 1 [file molecules-25-00335-s001.pdf]
